# Cultured cells activate IRE1 during attachment and flattening after routine passaging

**DOI:** 10.17912/micropub.biology.001968

**Published:** 2026-01-12

**Authors:** Paige Dillon, Lincoln Hollingshead, Julie Hollien

**Affiliations:** 1 School of Biological Sciences, University of Utah, Salt Lake City, Utah, United States

## Abstract

During endoplasmic reticulum (ER) stress, the ER membrane protein IRE1 initiates the regulated splicing of *Xbp1* mRNA, leading to the production of a potent transcription factor that helps cells restore proteostasis. We report that *Xbp1* is also spliced following the routine passaging of mouse MC3T3-E1 cells, without the addition of canonical ER stressors. This splicing was independent of the type of dissociation buffer used to release cells from the surface, but was reduced when cells were plated on non-adherent culture dishes. These findings suggest that certain cultured mammalian cells induce an unfolded protein response during reattachment and spreading after passaging.

**Figure 1. MC3T3-E1 cells splice Xbp1 following passaging:**
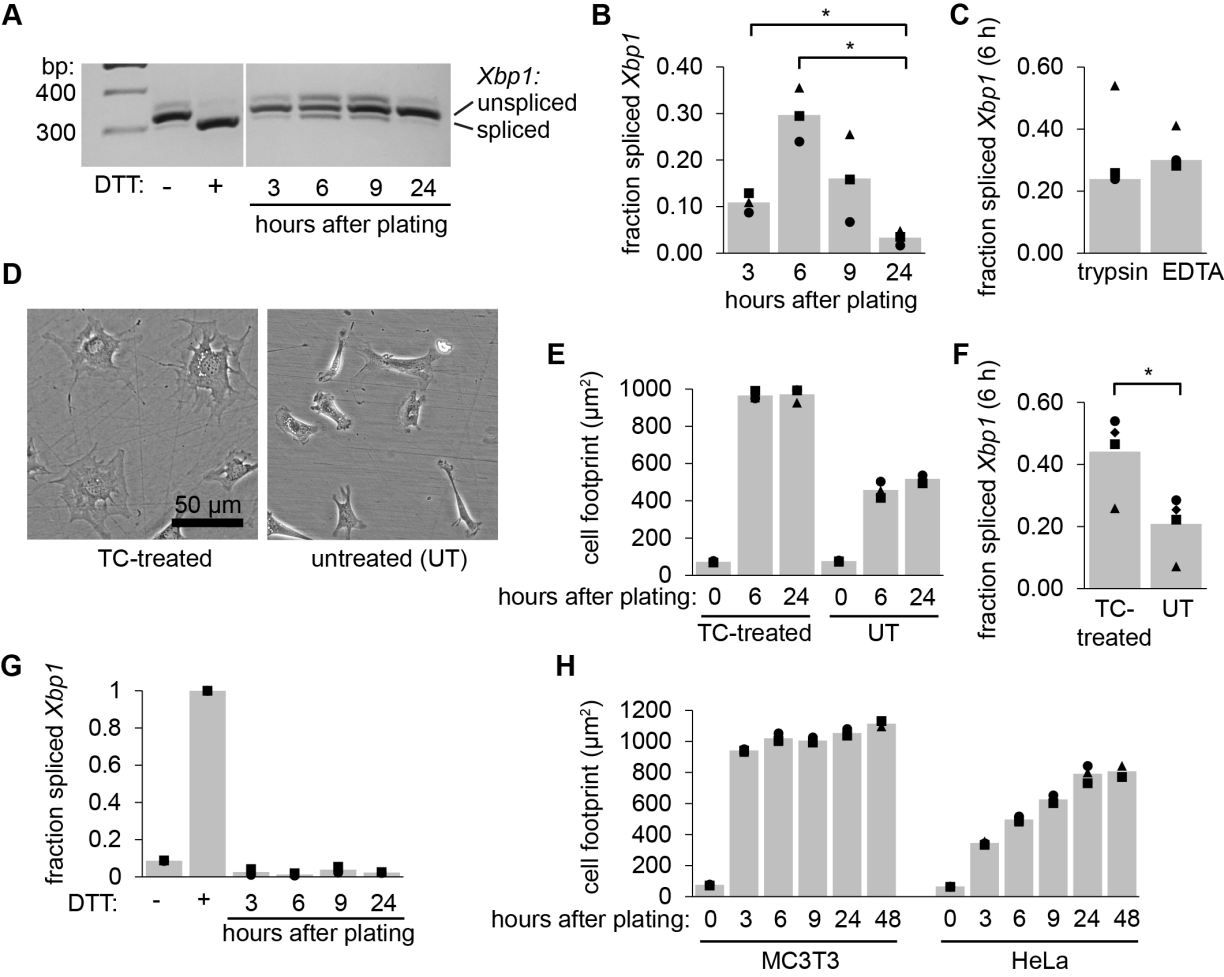
(A) We passaged MC3T3-E1 cells using trypsin-EDTA, collected RNA samples over time, and analyzed *Xbp1* splicing by RT-PCR and gel electrophoresis. DTT treatment was used as a control for ER stress. (B) Quantification of the fraction of *Xbp1* that was spliced following cell passaging in 3 replicate experiments. &nbsp;(C) Comparison of the fraction of spliced *Xbp1,* 6 h after passaging using trypsin vs non-enzymatic cell dissociation with EDTA. (D) Representative images of cells 6 h after passaging using trypsin and plated onto TC-treated vs untreated plates. (E) Quantification of the cell footprint (attached surface area) following passaging as in D. (F) Quantification of the fraction of spliced *Xbp1,* 6 h after passaging as in D. (G) We passaged HeLa cells, or treated with DTT as a control, and monitored *Xbp1* splicing as in A. (H) Quantification of the cell footprint of MC3T3 vs HeLa cells over time following passaging with trypsin. * indicates a p-value of < 0.05 as determined by paired t-tests followed by Holm-Bonferroni correction for multiple pairwise comparisons where appropriate.

## Description

IRE1 is an endoplasmic reticulum (ER) transmembrane resident protein and key mediator of the unfolded protein response (Walter and Ron, 2011; Wiseman et al., 2022). When activated by protein misfolding or lipid stress in the ER, the cytosolic-facing nuclease domain of IRE1 cleaves the mRNA encoding the transcription factor XBP1, initiating a noncanonical splicing event that leads to the downstream upregulation of many genes involved in protein folding, processing, and degradation (Yoshida et al., 2001; Calfon et al., 2002). We noticed an increase in *Xbp1* splicing following routine passaging of MC3T3-E1 cells, which are cultured fibroblasts derived from mouse calvaria, often used for microscopy due to their broad and flat morphology. This observation led us to investigate how passaging affects IRE1 activation in these cells.

We released MC3T3-E1 cells from their culture flasks using trypsin-EDTA, diluted in fresh media, replated cells in new flasks, and collected RNA after 3, 6, 9, and 24 hours. As a control, we treated cells with dithiotreitol (DTT, 2 mM, 6 h), a strong reducing agent and inducer of protein misfolding in the ER. We then purified total RNA, synthesized cDNA, used PCR to amplify *Xbp1* with primers surrounding the 26 nucleotide intron, and analyzed the products by agarose gel electrophoresis. We observed substantial *Xbp1* splicing, which peaked at 6 hours post-passaging and returned to baseline levels by 24 hours (A, B). This splicing did not appear to be a consequence of the proteolysis of plasma membrane proteins by trypsin, as we observed similar levels of splicing when we used a non-enzymatic, EDTA-only dissociation reagent (C).

Because *Xbp1* splicing was delayed following passaging and was not dependent on proteolytic dissociation methods, we wondered whether IRE1 activation was caused by reattachment of cells to the flasks. To test this, we compared cells plated onto standard TC-treated plates vs. untreated plates that do not promote attachment. Both *Xbp1* splicing (D) and the area of the cell footprint (E, F) were diminished by about 50% on the untreated plates. We conclude that MC3T3-E1 cells activate IRE1 while reattaching and spreading out following passaging.

&nbsp;To test whether other tissue culture cells also splice *Xbp1* after passaging, we carried out similar experiments in HeLa cells. HeLa cells did not splice *Xbp1* after passaging (G) and did not spread out as rapidly or to the same extent as MC3T3-E1 cells (H), again suggesting that the rapid attachment and spreading of MC3T3-E1 cells is the key event that leads to *Xbp1* splicing following passaging.

We speculate that *Xbp1* &nbsp;splicing may be caused by the increase in surface area and membrane tension as cells transition from a sphere-like morphology during dissociation to a flattened morphology when attaching to the substrate. The unfolded protein response can be activated by a variety of types of mechanical stress (Townson and Progida, 2025; Chen et al., 2025), and IRE1 is activated by saturated fatty acids and lipid bilayer stress resulting in increased membrane packing and thickness (Halbleib et al., 2017; Promlek et al., 2011; Thibault et al., 2012; Volmer et al., 2013). It remains unclear how changes in cell shape and attachment would propagate to the ER, but this could happen through ER-plasma membrane contact sites, through alterations in the cytoskeleton, or through disruptions to Ca ^2+^ homeostasis mediated by mechanically-gated ion channels. In any case, the unfolded protein response coordinates many aspects of lipid synthesis and metabolism (Moncan et al., 2021), which may help to re-establish homeostasis at the plasma membrane following the disruptions inherent to passaging cells. Our observations may therefore inform studies on the role of IRE1 and the ER in responding to mechanical stress, and may be generally useful to researchers studying ER stress in cultured cells.

## Methods

*Cell culture*

We cultured MC3T3-E1 cells in MEMα with nucleosides, L-glutamine, and no ascorbic acid (Life Technologies) supplemented with 10% FBS at 37 C and 5% CO _2_ . We cultured HeLa cells in DMEM supplemented with 10% FBS at 37 C and 5% CO _2_ . Both cell lines were purchased from American Type Culture Collection (ATCC).

*Xbp1 splicing assay*

We washed subconfluent MC3T3-E1 and HeLa cells with D-PBS (GenClone), treated with 0.05% trypsin-EDTA (Gibco) for 4 minutes, then diluted to a final cell count of 2-3×10 ^5^ in MEMα and plated in 25 cm ^2^ flasks. For nonenzymatic dissociation, we treated cells with Versene (Gibco) for 8 minutes before diluting in MEMα. For experiments comparing *Xbp1* splicing on treated and untreated plates, we used 10 cm, non-treated, sterilized VWR Tissue Culture Dishes (Avantar) along with treated 10 cm tissue culture dishes (Gen Clone). To collect RNA, we gently washed with PBS, then added 500 μL of RNA lysis buffer directly to the flasks or plates and purified total RNA using Quick RNA Miniprep kits (Zymo Research). Most cells remained attached to the surface during washing, and RNA yields were unchanged between 3 and 6 h after trypsinization, increasing by ~26% after 9 h and ~70% after 24 h, as expected based on their ~24-hour doubling time. Average RNA yield from untreated plates was ~82% of that from tissue-culture treated plates, suggesting that some cells were lost due to lack of attachment but not enough to explain the 2-fold difference in *Xbp1* splicing. We synthesized cDNA using Moloney murine leukemia virus reverse transcriptase (New England Biolabs) and T18 as a primer. We amplified cDNA with primers surrounding the regulated *Xbp1* splice site (mouse: AGAAGAGAACCACAAACTCCAG and GGGTCCAACTTGTCCAGAATGC or human: AGCTCAGACTGCCAGAGATCG and AATCCATGGGGAGATGTTCTG) and ran the PCR products on 2% agarose gels. We quantified the intensities of the spliced and unspliced *Xbp1* bands using ImageJ.

*Cell footprint calculations*

We imaged cells using an Amscope 4k HD408L camera and a cell-culture inverted microscope (Olympus CKX53, 10x, NA = 0.25). We collected measurements of the cell footprint, defined as the surface area occupied by a cell, by tracing 50 cells per condition. All experiments were repeated in triplicate and quantified using ImageJ.
